# Supplementary material for: Efficient solar hydrogen generation in microgravity environment
Source: Nat Commun. 2018 Jul 10;9:2527. doi: 10.1038/s41467-018-04844-y (PMC6039473; doi:10.1038/s41467-018-04844-y)
Supplement: Supplementary file 1 — Supplementary Information [file 41467_2018_4844_MOESM1_ESM.pdf]

Supplementary Information

Efficient Solar Hydrogen Generation in Microgravity Environment

Brinkert et al.

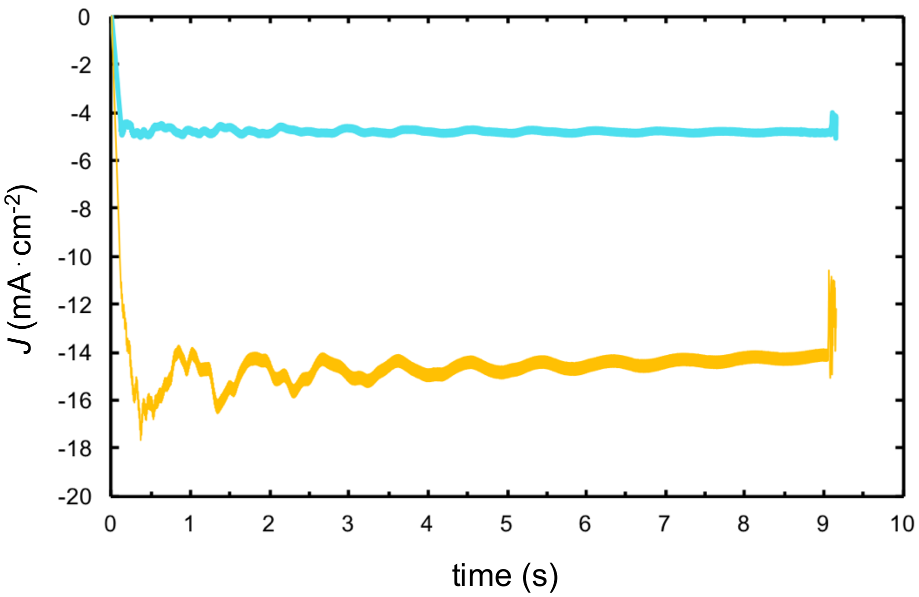

Supplementary Figure 1 | Chronoamperometric measurements in microgravity environments. Chronoamperometric measurement of the thin film (cyan) and nanostructured (yellow) p-InP-Rh photoelectrodes in microgravity environment (10^{-6} g) at 70 mW/cm^2 illumination with a W-I lamp in 1 M $HClO_4$ with the addition of 1 % (v/v) isopropanol at an applied potential of -0.09V vs. RHE. The applied potential was set to -0.09V vs RHE. The increased signal-to-noise ratio at the end of the measurements is due to the deceleration of the drop capsule after 9.3s.

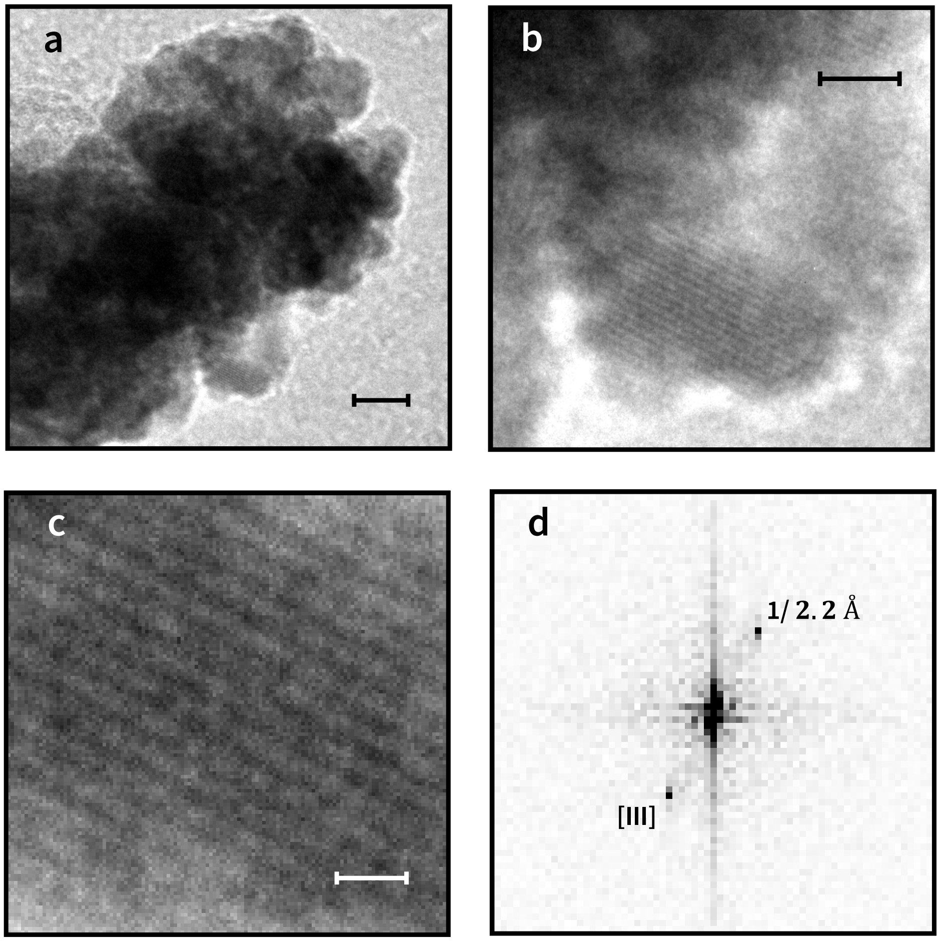

Supplementary Figure 2 | HRTEM images of the nanostructured photoelectrode. **a-c** HRTEM images of a rhodium grain from the nanostructured photoelectrode at different magnifications. The scale bars indicate the resolution of 5nm (**a**), 2nm (**b**) and 0.5 nm (**c**), respectively. **d** 2D-Fourier Transformation image showing the corresponding diffraction pattern with a lattice plane spacing of 2.2 Å hkl 111, which is typical for cubic structures.

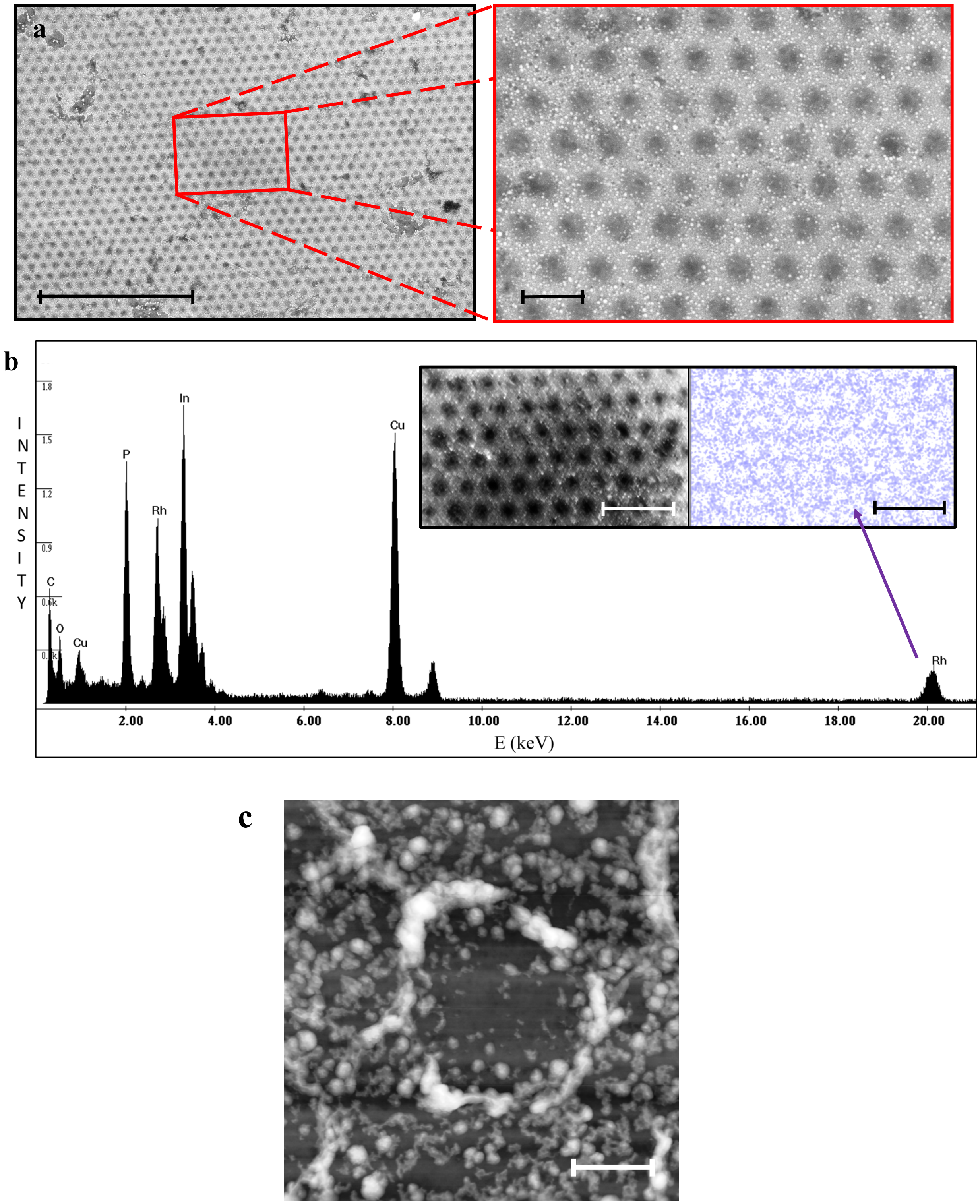

Supplementary Figure 3 | SEM and AFM images of the nanostructured photoelectrode. **a** SEM image demonstrating the periodic structure of the nanostructured photoelectrode. The scale bars indicate the resolution of 10μm and 1μm, respectively. **b** EDXS spectrum showing the element composition of the

photoelectrode sample. Inset of a SEM image shows that Rh corresponds to the periodic structure. For obtaining the spectra, the electrode was kept assembled. The Cu wire attached to the ohmic back contact of the p-InP gives rise to the Cu signal. The quantitative EDAX analysis was carried out for Rh at different points on the samples. The inset bright field image was taken under the condition of minimum phase contrast [1], showing the periodical distribution of Rh nanocrystals. The scale bars indicate the resolution of 2µm. **c** Tapping mode AFM image of the nanostructured catalytic layer of rhodium on p-InP at a resolution of 500nm, indicated by the scale bar.

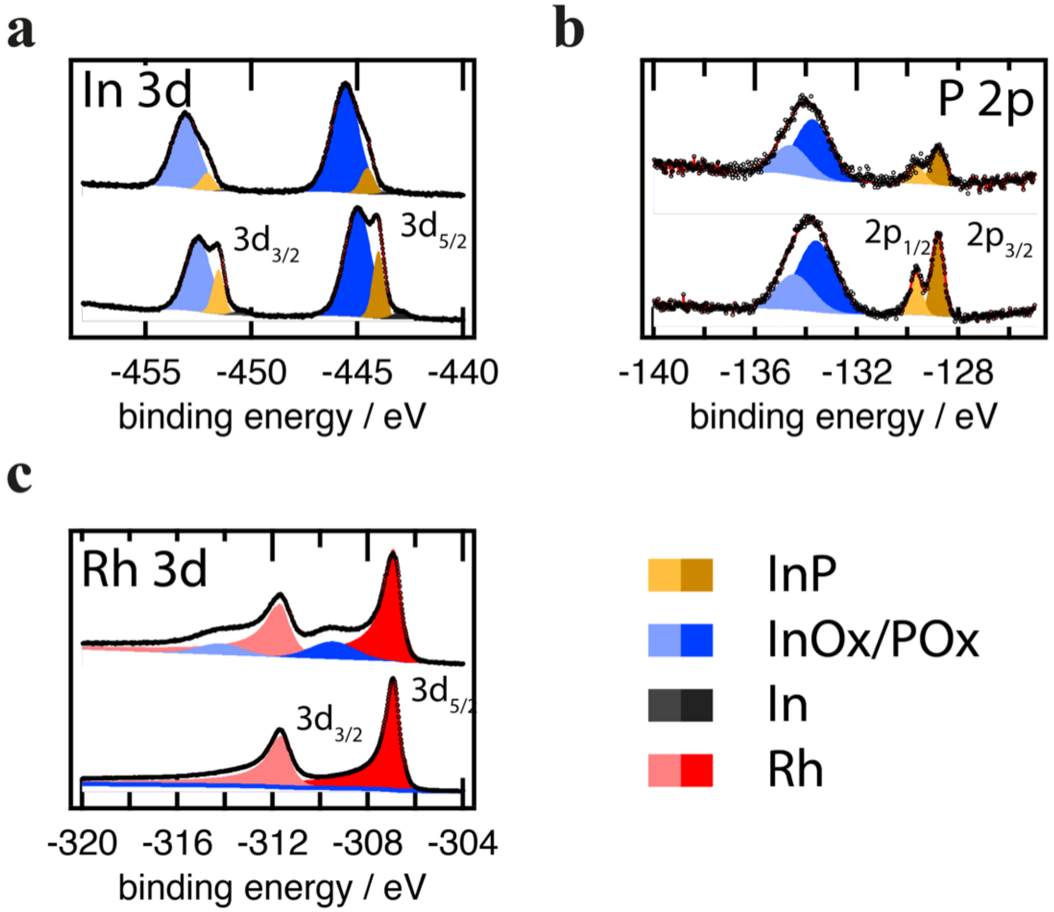

Supplementary Figure 4 | X-ray photoelectron spectra of the thin film and nanostructured photoelectrodes. X-ray photoelectron spectra of the nanostructured (top) and thin film (bottom) p-InP-Rh photoelectrodes. **a** In 3d core levels; **b** P 2p core levels and **c**) Rh 3d core levels. The color coding under the lines refers to the respective composition as illustrated in the legend.

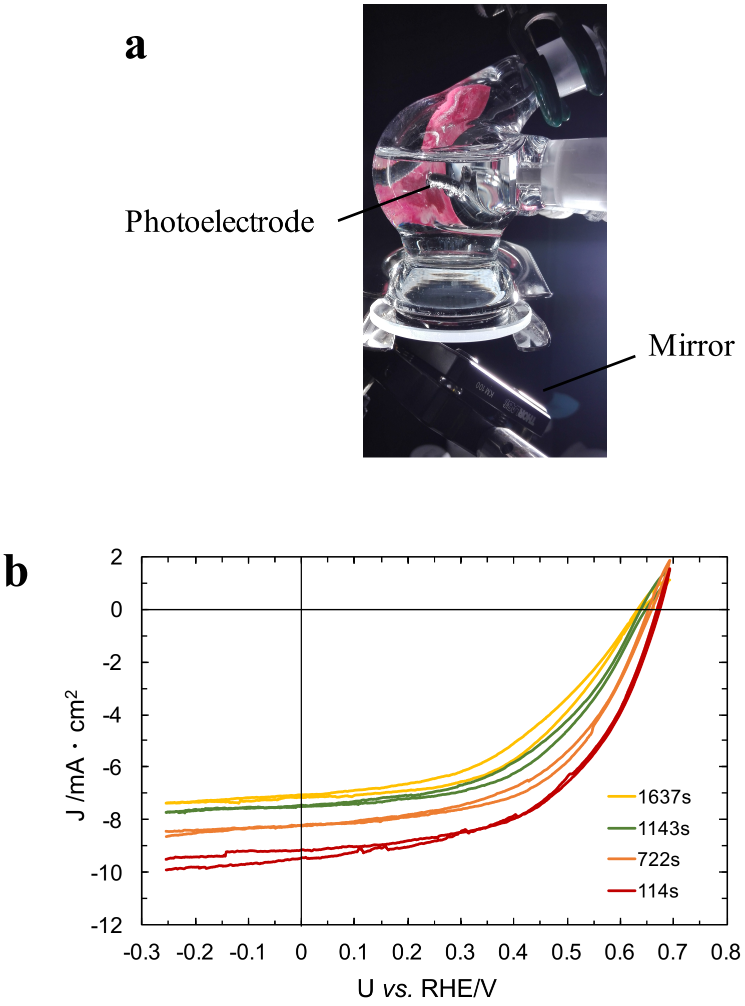

Supplementary Figure 5 | Terrestrial experiments demonstrating the effect of mass transfer limitations on the J-V behaviour. **a** Experimental set-up for investigating the influence of mass transfer limitations on the J-V characteristics of a photoelectrochemical cell, mimicking microgravity conditions terrestrially. The photoelectrochemical cell was placed upside down, illumination (70mW/cm^2, white-light 1000W Mercury-Xenon arc lamp with a calibrated AAA grade AM1.5G filter) of the thin-film photoelectrode occurred through a mirror into the glass cell. **b** J-V measurements of the thin film photoelectrode in the set-up described in (**a**). The scan rate was set to 50mV/s. Selected measurements are shown at indicated time points. Short circuit current and open circuit voltage decrease with progressing light-induced hydrogen production due to 'trapped' hydrogen gas bubbles on the electrode surface. After the last measurement at 1637s (yellow curve), the initial J-V curve (red) could be recovered again by addition of 1% (v/v/) isopropanol to the electrolyte.

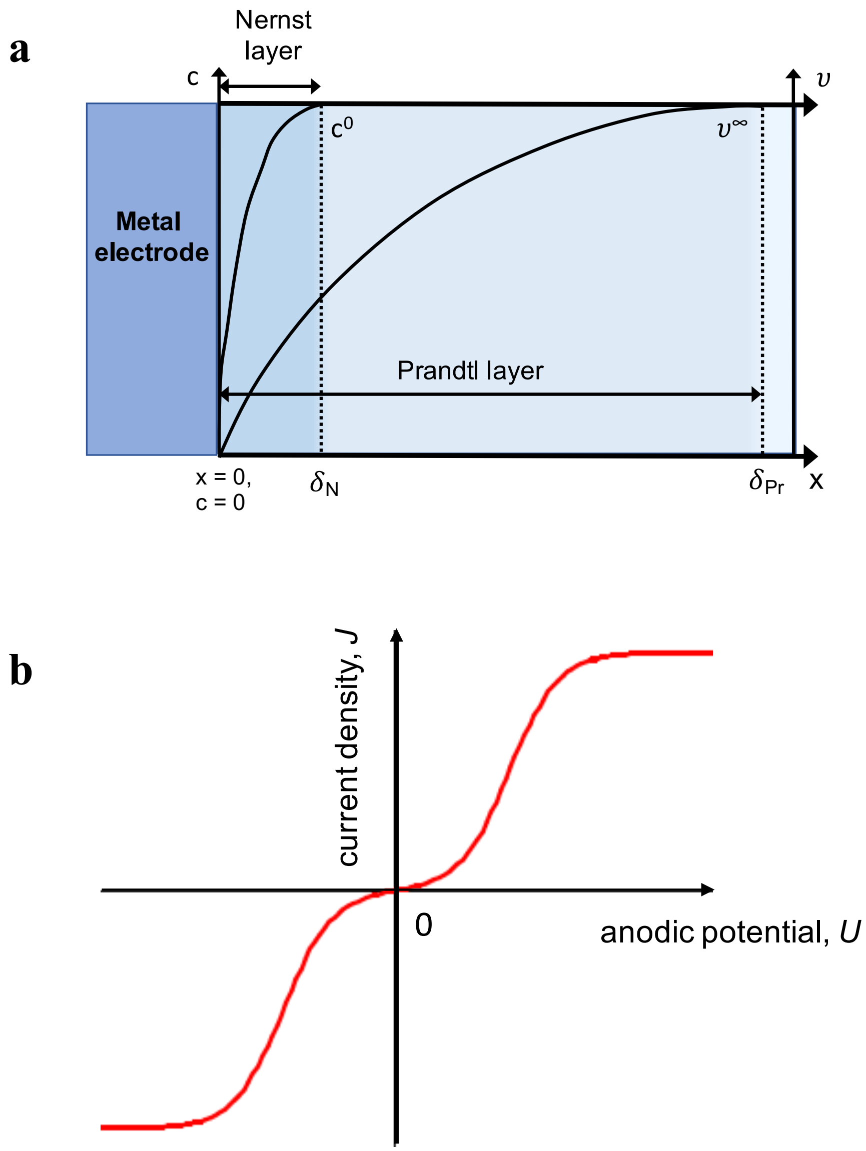

Supplementary Figure 6 | Illustration of the Butler-Volmer equation for diffusion limitation. **a** Illustration of the Nernst diffusion layer, δ_N, and the Prandtl layer, δ_{Pr}: c^0 is the solution concentration of the bulk and υ^∞ is the solution velocity at (theoretically) infinite distance from the electrode. **b** J-V characteristics of the Butler-Volmer equation for diffusion limitation.

Supplementary Table 1 | Experimental sequence of photoelectrochemical hydrogen production at the Bremen Drop Tower. Detailed experimental sequence for photoelectrochemical hydrogen production hydrogen production in microgravity environment. For a compact illustrative graphic, please see **Figure 1** in the main paper.

Time	Experimental sequence
-120 min	Photoelectrodes are placed in PEC cell set-up, capsule is closed with cover
-105 min	Capsule is transported into drop tower, attachment to catapult
-90 min	Start of drop tube evacuation; capsule is purged with argon
-12 min	Evacuation is stopped, removal of power supply to capsule, catapult pulls capsule onto start position
-10.31 s	**Start programmed drop sequence**
-0.31 s	Cameras turn on, samples are immersed into electrolyte
-0.01 s	Lights turn on
0 s	Capsule is released from catapult, microgravity environment enabled
0.02 s	Potentiostats turn on
	Experiment starts
9.32 s	Capsule decelerates in container, microgravity environment disabled
11.32 s	Experiment stops; lights turn off
12.32 s	Samples are emersed from electrolyte
19.32 s	Potentiostats and cameras turn off, **programmed drop sequence stops**
15 min	Capsule is lifted up from deceleration container
20 min	Capsule cover is removed, sample is no longer under argon; capsule is transported back to docking station where instruments are connected again to power supply
25 min	Samples are rinsed with MiliQ water, dried under nitrogen and transferred into the glove box for temporary storage

Supplementary Table 2 | Terrestrial open circuit potentials of the thin film and nanostructured photoelectrode. Open circuit potentials (V_{OC}) of five independent cyclic voltammetry measurements (terrestrial conditions, light intensity 70mW/cm^2) with five thin film and nanostructured photoelectrodes in 1M $HClO_4$. Scan rate: 50mV/s, scan range: -0.1V to +0.65V vs RHE. The single measurements indicate that the V_{OC} of the thin film and nanostructured electrodes are almost identical.

	**V_{OC} thin film [V]**	**V_{OC} nanostructured [V]**
	0.5596	0.6316
	0.5809	0.5880
	0.5934	0.5840
	0.6008	0.5299
	0.5022	0.6030
**average**	**0.5674**	**0.5873**
**standard deviation**	0.0396	0.0371

Supplementary Note 1.

Derivation of the diffusion limited current

Under certain conditions, the flux of an electroactive substance at the electrode surface controls the rate of reaction and therefore, the faradaic current flowing in the external circuit. The total current density is given by the sum of diffusion, convection and migration:

$$j_t = j_{diff} + j_{con} + j_{mi} \quad (1)$$

Assuming that field-assisted transport (migration) can be neglected, diffusion is the dominating process, supported by either natural or forced convection. Under ideal conditions, the reaction is limited by the rate of catalysis and these transport effects can be neglected because the concentrations at the electrode surface and in the bulk of the solution equilibrate instantaneously. Under microgravity conditions, however, a limiting current was obtained for the thin film sample that was much lower than its limiting current under terrestrial conditions, suggesting that the rate-limiting step for the electrochemical reaction was different. This leads to the conclusion that

(i) The electrode reaction is faster than the ion transport to the surface

(ii) At the surface, reactants are depleted and the products accumulate for semiconductor-based systems, this also results in increased recombination of light-induced excess minority carriers which is not mathematically included in this consideration

(iii) The rate of reactant diffusion to the surface is determining the current density.

We can describe the rate of diffusion to the surface assuming a Nernst diffusion layer with thickness δ_N (a boundary layer): hence, the current density can be determined using Fick's 1st law. In a cathodic net reaction, A (H_2) is produced at the electrode surface, so that c_A^0 (x = 0, electrode surface) > c_A (bulk). The corresponding current density is then given by:

$$j = nF\frac{D_A}{\delta_N}(c_A^0 - c_A) \quad (2)$$

D_A is the diffusion constant for hydrogen. Accordingly, the mass-transport limited maximum current density, j_{mtl}, is defined as:

$$j_{mtl} = nF \frac{D_A}{\delta_N} c_A^0 \qquad (3)$$

The ratio of the concentrations can be expressed by the two currents:

$$\frac{c_A^0}{c_A} = 1 - \frac{j}{j_{mtl}} \qquad (4)$$

The concentration ratio can be inserted into the cathodic and anodic branches of the Butler-Volmer equation, respectively, yielding the result shown in SI Fig. 6 a):

$$j_{mtl,c} = j_0 \left[\frac{c_O^0}{c_O} \cdot \exp\left(\frac{-\alpha neV}{kT} \right) \right] \qquad (5a)$$

$$j_{mtl,a} = j_0 \left[\frac{c_R^0}{c_R} \cdot \exp\left(\frac{(1-\alpha)neV}{kT} \right) \right] \qquad (5b)$$

Here, j_0 denotes the exchange current density, α the charge transfer coefficient, V is the overpotential with regard to the thermodynamic equilibrium and n is the number of electrons transferred which is 2 in this case. Taking eqns.(5a) and (5b) that show the Butler-Volmer equation for mass transport limitation, one obtains the J-V characteristic:

$$\frac{j}{j_0} = \left(1 - \frac{j}{j_{mtl,c}}\right) \cdot \exp\left(\frac{-\alpha neV_{mt}}{kT} \right) - \left(1 - \frac{j}{j_{mtl,a}}\right) \cdot \exp\left(\frac{(1-\alpha)neV_{mt}}{kT} \right) \qquad (6)$$

A full derivation of eqn.(6) from eqn.(5a) and (5b) can be found in Bard and Faulkner (2001) [2]. The first term describes the cathodic current density, the second term the anodic one. V_{mt} is the overpotential due to mass transport. Solving for the current density, one obtains a modified Butler-Volmer eqn.:

$$j(V_{mt}) = \exp\left(\frac{-\alpha neV_{mt}}{kT}\right) - \exp\left(\frac{(1-\alpha)neV_{mt}}{kT}\right) \cdot \left(\frac{1}{j_0} + \frac{1}{j_{mtl,c}} \cdot \exp\left(\frac{-\alpha neV_{mt}}{kT}\right) - \frac{1}{j_{mtl,a}} \cdot \exp\left(\frac{(1-\alpha)neV_{mt}}{kT}\right)\right)^{-1} \quad (7)$$

If the exchange current becomes infinite, the measured current density is controlled by diffusion; the left-hand side of eqn. (7) vanishes and from

$$0 = \left(1 - \frac{j}{j_{mtl,c}}\right) \cdot \exp\left(-\frac{\alpha neV}{kT}\right) - \left(1 - \frac{j}{j_{mtl,a}}\right) \cdot \exp\left(\frac{(1-\alpha)neV}{kT}\right) \quad (8)$$

one obtains the mass transfer limited current density:

$$j_{mtl}(V_{mt}) = \left(1 - \exp\left(-\frac{neV_{mt}}{kT}\right)\right) \cdot \left(\frac{1}{j_{mtl,a}} - \frac{1}{j_{mtl,c}} \cdot \exp\left(-\frac{neV_{mt}}{kT}\right)\right)^{-1} \quad (9)$$

Eqn. (9) was used here to determine the current-voltage curve under mass transport limitation (see main article for details).

Supplementary References

[1] Kunath, W., Zemlin, F., Weiss, K. Apodization in phase-contrast electron microscopy realized with hollow-cone illumination. *Ultramicroscopy* **16** (2), 123-138 (1985).

[2] Bard, A. L. and Faulkner, L. R. Electrochemical Methods: Fundamentals and Applications. 2nd edition, New York: Wiley (2001).
